# Interventions to Improve Antiretroviral Therapy Adherence Among Adolescents and Youth in Low- and Middle-Income Countries: A Systematic Review 2015–2019

**DOI:** 10.1007/s10461-020-02822-4

**Published:** 2020-03-09

**Authors:** Lindsey K. Reif, Elaine J. Abrams, Stephen Arpadi, Batya Elul, Margaret L. McNairy, Daniel W. Fitzgerald, Louise Kuhn

**Affiliations:** 1 Center for Global Health, Department of Medicine, Weill Cornell Medicine, New York, NY USA; 2 Gertrude H. Sergievsky Center, Vagelos College of Physicians and Surgeons, Columbia University Irving Medical Center, New York, NY USA; 3 ICAP At Columbia University, Mailman School of Public Health, Columbia University Irving Medical Center, New York, NY USA; 4 Department of Pediatrics, Vagelos College of Physicians and Surgeons, Columbia University Irving Medical Center, New York, NY USA; 5 Division of General Internal Medicine, Department of Medicine, Weill Cornell Medicine, New York, NY USA; 6 Department of Epidemiology, Mailman School of Public Health, Columbia University Irving Medical Center, New York, NY USA

**Keywords:** Adolescent, Youth, ART adherence, Review, Intervention

## Abstract

Adolescents and youth living with HIV have poorer antiretroviral treatment (ART) adherence and viral suppression outcomes than all other age groups. Effective interventions promoting adherence are urgently needed. We reviewed and synthesized recent literature on interventions to improve ART adherence among this vulnerable population. We focus on studies conducted in low- and middle-income countries (LMIC) where the adolescent and youth HIV burden is greatest. Articles published between September 2015 and January 2019 were identified through PubMed. Inclusion criteria were: [1] included participants ages 10–24 years; [2] assessed the efficacy of an intervention to improve ART adherence; [3] reported an ART adherence measurement or viral load; [4] conducted in a LMIC. Articles were reviewed for study population characteristics, intervention type, study design, outcomes measured, and intervention effect. Strength of each study’s evidence was evaluated according to an adapted World Health Organization GRADE system. Articles meeting all inclusion criteria except being conducted in an LMIC were reviewed for results and potential transportability to a LMIC setting. Of 108 articles identified, 7 met criteria for inclusion. Three evaluated patient-level interventions and four evaluated health services interventions. Of the patient-level interventions, two were experimental designs and one was a retrospective cohort study. None of these interventions improved ART adherence or viral suppression. Of the four health services interventions, two targeted stable patients and reduced the amount of time spent in the clinic or grouped patients together for bi-monthly meetings, and two targeted patients newly diagnosed with HIV or not yet deemed clinically stable and augmented clinical care with home-based case-management. The two studies targeting stable patients used retrospective cohort designs and found that adolescents and youth were less likely to maintain viral suppression than children or adults. The two studies targeting patients not yet deemed clinically stable included one experimental and one retrospective cohort design and showed improved ART adherence and viral suppression outcomes. ART adherence and viral suppression outcomes remain a major challenge among adolescents and youth. Intensive home-based case management models of care hold promise for improving outcomes in this population and warrant further research.

## Introduction

Adolescents and youth, 10 to 24 years of age, represent a growing proportion of people living with HIV around the world and have worse outcomes than all other age groups [1–6]. In recent years, AIDS-related deaths among adolescents and youth increased by 50% while they have decreased among all other age groups [7]. In 2018, 510,000 young people between the ages of 10 to 24 years were newly-infected with HIV, 40% of whom were between 10 and 19 years of age [8]. In addition to heterosexual transmission, a generation of children infected with HIV perinatally are now aging into adolescence, adding to the burden of disease in this age group.

Adequate adherence to an antiretroviral therapy (ART) regimen leading to viral suppression is essential for an adolescents’ own health and well-being, and to reduce further HIV transmission. Yet, adolescents and youth have poor adherence to drug regimens for many chronic illnesses [9–12]. Adherence to ART is further complicated by HIV-related stigma [13–16]. The period of adolescence and youth is characterized as a time of great physiological and psychological growth and development [1, 17], increased desire for independence from parents [18], and increased risk-taking [19], adding another layer of complexity. During this developmental stage, initiation of sexual activity is common and may include early pregnancy [20]. Adolescents and youth also lack financial autonomy, are prone to peer pressure, and lack problem-solving skills [21–25]. Further, in resource-limited settings, external factors including poverty, food scarcity, and HIV-related stigma acutely influence ART adherence and HIV outcomes [26–33].

Major barriers to ART adherence for adolescents and youth can be divided into 3 categories: patient-level factors (e.g. socioeconomic status, stigma) [34, 35], health services factors (e.g. clinic waiting times, drug availability, quality of care) [36, 37], and medication factors (e.g. dosing, high pill burden, side effects) [3, 38, 39]. Much of the research on ART adherence among young people has focused on identifying and estimating the prevalence of these barriers [29, 33, 38, 40, 41]. A comprehensive review of the literature between 2003 and 2015 identified 10 studies which evaluated interventions to improve adherence in adolescents in developed countries [42]. Effective interventions included daily interactive text reminders for dosing [43, 44], and computer-driven support programs [45]. However, none of the studies included were conducted in a low- or middle-income country (LMIC) [46], areas where the global HIV epidemic is centered. Further, most of these studies were descriptive reports or pilot studies with small sample sizes and thus had insufficient power to detect meaningful effects.

Given the critical need to identify effective approaches to improve outcomes among adolescents and youth living with HIV, we evaluated and synthesized the recent published literature on research conducted in a LMIC aimed at improving ART adherence in this population.

## Methods

### Article Search and Selection

**Fig. 1:**
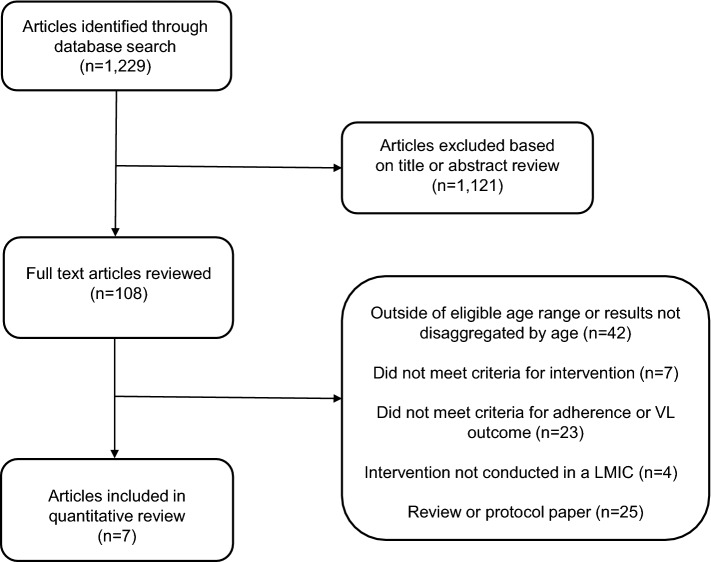
Flowchart for articles published in PubMed between September 2015 and January 2019 which were reviewed under search term [(((((((structural) or ((behavioral))) and intervention)) and ((((((ART) or HIV medication) or HAART)) and ((adherence) or persistence)) and HIV))) and (((young adult) or adolescent) or adolescence) or youth))]

We searched the PubMed database for English language articles which evaluated interventions to improve ART adherence among adolescents and youth living with HIV, conducted in a LMIC, and published between September 2015 and January 2019 using the search terms indicated in Fig. 1 [47]. We then manually reviewed the references sections of relevant articles. Records were managed using EndNote and duplicates were removed manually. One reviewer (LR) conducted the primary search and articles selected for inclusion were approved by all authors. Articles selected met the following criteria: (1) included adolescents and youth ages 10–24 years; (2) evaluated an intervention to improve ART adherence; (3) included an ART adherence measurement outcome (self-report, pill count, or medication event monitoring systems (MEMS)) or viral load (VL) as a proxy for an adherence measurement; (4) conducted in a LMIC. Eligible manuscripts were not restricted by study design and included studies which evaluated structural, behavioral, or health services-related interventions aimed at improving ART adherence.

### Data Extraction and Synthesis

Each of the articles meeting our inclusion criteria was reviewed for characteristics of the study population (e.g. age, time on ART, health status at baseline), type of intervention evaluated, study design, outcome measured (ART adherence or VL), and intervention efficacy. The strength of each study’s evidence was evaluated according to an adapted GRADE system utilized by the World Health Organization [48]. This system classifies studies into four levels based on study design, analysis plan, and existence of comparison groups. High quality evidence (level 4) are randomized control trials (RCT) with statistical testing comparing groups. Moderate quality evidence (level 3) are prospective cohort studies with statistical testing comparing groups. Low quality evidence (level 2) are retrospective or descriptive studies with statistical testing of between or within group comparisons; and very low quality evidence (level 1) are studies without statistical comparisons.

Due to the small selection of studies and diversity of outcome measures, a meta-analysis was not conducted to synthesize the effect size across studies. We reviewed the seven included studies to determine interventions with the most potential for impact and assessed areas in need of further research and evidence.

## Results

### Included Studies and Populations

The PubMed search identified 1229 articles, of which 1121 were excluded based on review of the abstract. A full text review was completed on 108 articles. Seven studies met the inclusion criteria. Reasons for exclusion are listed in Fig. 1; if an article was excluded for failing to meet more than one criteria, it is listed under the first exclusion category as prioritized in the criteria above. The most common reason for exclusion was only including participants outside the age range of interest (10–24 years) or reporting outcomes which were not disaggregated so as to discern results among adolescents and youth (i.e. results reported for ages 18–29 years). The seven studies meeting our inclusion criteria are described in Table 1. All were conducted in sub-Saharan Africa and included adolescents living with HIV who were aware of their HIV status and were receiving HIV care at a health facility. The median sample size was 702 (range 94–96,706).

**Table 1:** Evaluations of Interventions to Improve ART adherence among adolescents and youth ages 10–24 years

Study/country	Study population*	Intervention	Study design/outcome	Adherence measurement	Result	Level of evidence	Effective
Patient-specific interventions							
Linnemayr [51] Uganda	N = 332 Ages: 15–22 Baseline status: In HIV care ART-experienced	Control arm: Standard of care (no text messaging) 1 way arm: Received text messages only 2-way arm: Received text messages and could respond by text message Once weekly, for one year, participants in the 1-way arm received the message: “we hope you are feeling well today”; participants in the 2-way arm received the same message with the addition: “reply 1 if well, 2 if unwell”. Participants in the 2-way arm who did not respond or responded they were unwell received a follow-up message or a call from a study coordinator, respectively	3 arm individual RCT Outcome: Mean adherence over 48 weeks	Medication event monitoring system (MEMS)	Mean adherence between enrollment and 48 weeks: Control arm: 67.0% 1-way text arm: 64.0% 2-way text arm: 61.0% No significant differences	High quality	No
Bermudez [52] Uganda	N = 702 Ages: 10–16 Baseline status: In HIV care ART-experienced	A child savings account matched at a rate of 1:1 could be used on medical expenses, family business development, or education (school lunches and fees) was provided to a family. Participants also attended 4 workshops on financial management, and life skills (e.g. asset building, goal setting, and risk mitigation)	RCT Outcome: VL < 40 copies/µl	Viral load	Proportion of participants with VL < 40 copies/µl at baseline: Control arm: 62.2% Intervention arm: 55.0% (p = 0.18) Proportion of participants with VL < 40 copies/µl at 24 months: Control arm: 63.4% Intervention arm: 65.9% No significant difference	High quality	No
Nasuuna [53] Uganda	N = 192 Ages: 10–19 Baseline status: In HIV care ART-experienced VL > 1000 copies/μl	Nurses, adherence counselors or expert patients administered 3 consecutive monthly Intensive Adherence Counseling (IAC) sessions to identify barriers and intervene to improve adherence	Retrospective cohort Outcome: VL < 1000 copies/µl	Viral load	Proportion of participants with VL < 1000 copies/µl within 180 days of completing 3 IAC sessions: 117/192 (61%) of participants received a VL test after completing 3 IAC sessions. Results for these 117 include: Ages 10–14: 34.0% (26/77) Ages 15–19: 20.0% (8/40)	Very low quality	No

Health services interventions							
Kim [54] Botswana Lesotho Swaziland Malawi Uganda Tanzania	N = 5,008 Ages: 10–19 Baseline status: In HIV care ART-experienced ART adherent (pill count > 95%)	Participants received multi-month ART prescriptions (MMP), which would last between 2 and 6 months in order to reduce the frequency of required clinic visits for ART refills. Start of MMPs occurred when participants were deemed clinically stable (defined as improving CD4 cell count/CD4% or VL suppression, or minimal HIV-associated morbidity) and ART adherent (defined as pill count measured at 95–105%)	Retrospective cohort Outcome: VL < 400 copies/µl	Viral load	Proportion of participants with VL < 400 copies/µl by age at initiation of MMPs: Baseline: Ages 1–9: 85.0% Ages 10–19: 80.0% 60 months: Ages 1–9: 85.0% Ages 10–19: 75.0%	Low quality	No
Grimsrud [55] South Africa*	N = 884 Ages: 16–24 Baseline status: In HIV care ART-experienced 2 consecutive VL < 400 copies/μl	Community-based adherence clubs are groups of 25–30 participants who met every 2 months for group counseling, a symptom screen, and distribution of pre-packed ART. The groups were supported by a community health worker with a nurse available for necessary phlebotomy (CD4, viral load, creatinine as necessary) and clinical consultation	Retrospective cohort Outcome: (1) Retained in care (2) VL < 400 copies/µl	Viral load	Proportion of participants retained in care at 12 months by age: Ages 16–24: 90.9% Ages > 25: 94.1% (p = 0.022) Among those retained in care, proportion of participants with VL < 400 copies/µl at 12 months by age: Ages 16–24: 97.2% Ages > 25: 98.0% (p = 0.194)	Low quality	No
Fatti [56] South Africa	N = 6706 Ages: 10–24 Baseline status: Newly diagnosed with HIV ART-naïve	Community-based support workers are lay health workers who conducted home visits to identify and address participants’ challenges with maintaining retention and adherence, and offer caregivers education and information as necessary. Support workers were assigned to a patient at HIV testing and stayed through long-term care with weekly visits for the first several months following ART initiation, then monthly for 6 months, then quarterly. More frequent visits commenced if clinic visits lapsed	Retrospective Cohort Outcome: (1) Mean medication possession ratio (MPR) (2) VL < 400 copies/µl	Pill count and viral load	Participant adherence (MPR) at 5 years: Participants with a support worker: 82.5% Participants without a support worker: 83.0% No significant difference Proportion of participants with VL < 400 copies/µl at 5 years: Participants with a support worker: 81.2% Participants without a support worker: 62.8% (p = 0.055)	Moderate quality	Yes
Willis [57] Zimbabwe	N = 94 Ages 10–15 Baseline status: In HIV care ART-experienced	Community Adolescent Treatment Supporters are adolescents and youth ages 18 to 24 living with HIV, trained as peer counselors, who made weekly home visits to monitor well-being, provide adherence counseling and psychosocial support, and give caregivers information and counseling as necessary. Participants were also encouraged to participate in monthly support groups at a health facility and offered a pill box	RCT Outcome: self-reported ART adherence	Self-reported ART adherence	Proportion of participants self-reporting adherence at baseline: Control arm: 48.9% Intervention arm: 44.2% Proportion of participants self-reporting adherence at 12 months: Control arm: 39.3% Intervention arm: 71.8% (OR 3.93; 95% CI 1.40–11.02; p < 0.05)	High quality	Yes

^*^If a study included children and/or adults, we report the total number of adolescents and youth included, the age group specified, and results specific to this age group

We also reviewed and report the results of 4 articles that were excluded because the studies were not conducted in a LMIC. We briefly summarize the results of these studies here and their potential transportability to an LMIC setting, but did not include them in the main review since our primary goal was to identify interventions which would be directly applicable in resource-limited settings where the global HIV burden among adolescents and youth is greatest.

### Intervention Types

Studies either focused on: (1) patient-level interventions, i.e. interventions implemented at the individual level in addition to standard HIV care (n = 3); or (2) health services interventions which re-structured the way HIV care was provided, also called ‘models of HIV care’ [49, 50] (n = 4).

In the first patient-level intervention, a once-weekly SMS text message was designed to check-in with participants about their general well-being [51]. One study arm received this weekly check-in message but could not respond to the sender. The second arm received the same message and could respond to the sender. The third study arm received standard care, i.e. no messages. The second study evaluated an economic intervention in which a savings account was established which could be used for small business development or education, i.e. school fees or lunches to address financial-related barriers to ART adherence [52]. In the third study, three consecutive monthly individual intensive adherence counseling sessions were provided to participants. The goal of the sessions was to identify adherence barriers and develop individualized plans to address them [53].

**Fig. 2:**
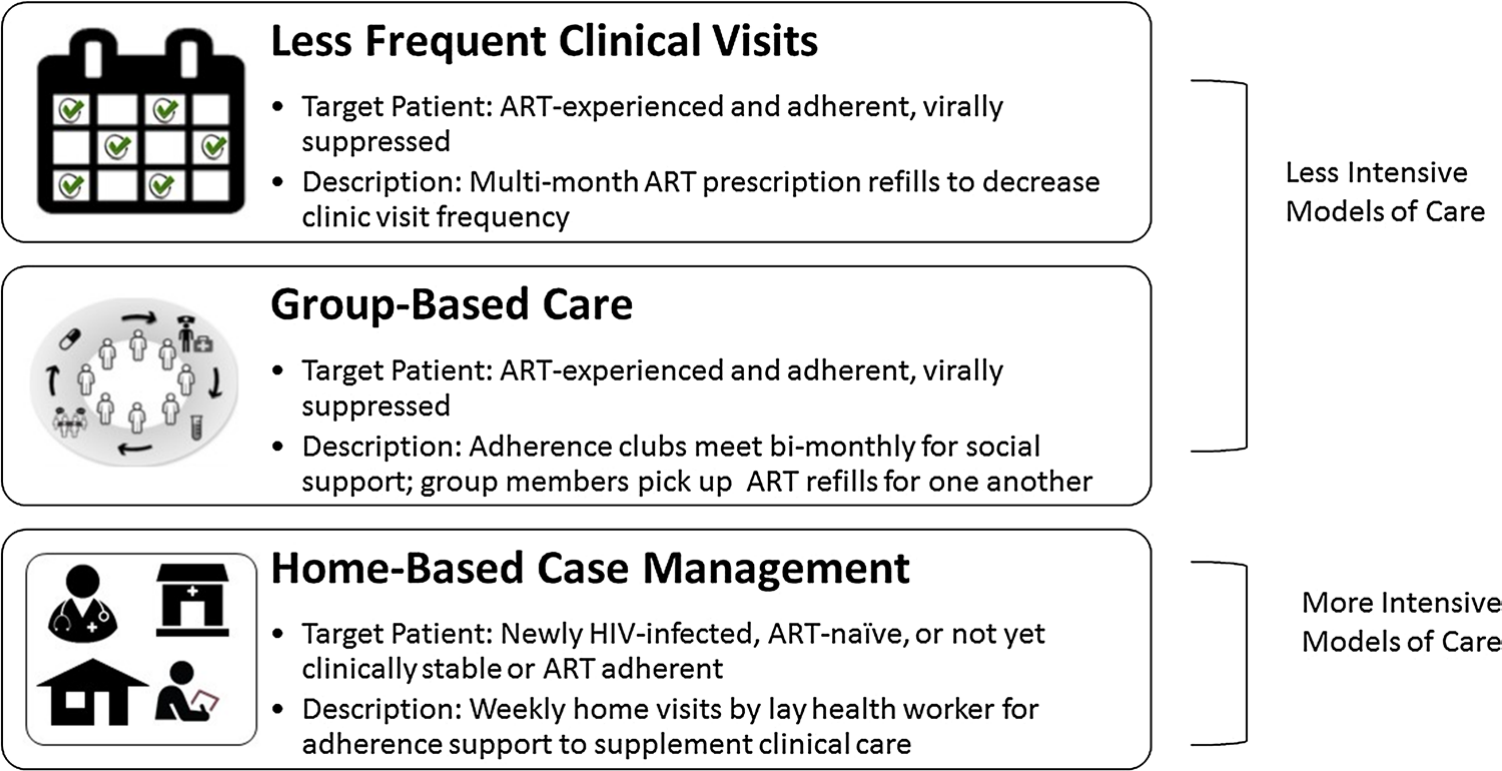
Types of health services interventions

Four studies evaluated health services interventions which are categorized as either less intensive (n = 2) or more intensive (n = 2) models of care (Fig. 2). The two less intensive models of care reduced the amount of time stable participants spent in the clinic. In the first, multi-month ART prescriptions allowed participants to come to the clinic less frequently ranging from every other month and some only twice per year for medication refills and clinical check-ups [54]. In the second, a group-based model of care, patients formed groups of 25–30 participants and met every other month for group counseling, brief check-ups, and distribution of ART refills [55]. Groups were facilitated by a lay health worker and nurse and met at a health facility or community venue.

Two studies of more intensive models of care targeted newly diagnosed adolescents initiating ART or those who had not yet been defined as adherent or stable. These two models of care augmented regular care with additional support through home-based case management by a community health worker [56] or peer counselor [57]. In the first, a community-based support worker (i.e. lay health worker) made weekly home visits to provide additional individualized adherence support. As participants became stable, frequency of home visits decreased to monthly and then quarterly, but increased again in the event of any clinic visit lapses [56). In the second, peer counselors (i.e. trained adolescents and youth ages 18 to 24 years living with HIV) referred to as ‘community adolescent treatment supporters’ provided adherence counseling and psychosocial support via weekly home visits [57].

### Patient-Specific Interventions

#### Study Design, Outcomes, and Participant Eligibility

Of the three studies which evaluated patient-level interventions, two used experimental designs and one entailed a retrospective cohort study. The study evaluating text messaging used a 3-arm RCT to compare text messaging and text messaging with the option to respond to standard care (no text messaging). The primary outcome was mean ART adherence over the 48-week study period which was measured using medication event monitoring systems (MEMS) and defined as the ratio of recorded bottle openings to the number of prescribed bottle openings [51]. Eligible patients were 15 to 22 years of age, in care at an HIV care facility, and prescribed ART. The study evaluating a participant savings account used a 2-arm RCT comparing the intervention to standard care. The primary outcome was VL < 40 copies/µl 24 months after study enrollment [52]. Eligible patients were ages 10 to 16 years of age, in care at an HIV care facility, and prescribed ART. The study evaluating monthly intensive adherence counseling sessions used a retrospective cohort design and no comparison group. The primary outcome was VL < 1000 copies/µl measured within 180 days of completing the 3 sessions [53]. Eligible patients were ages 10 to 19 years of age, in care at an HIV care facility, prescribed ART and had a VL > 1000 copies/µl.

#### Intervention Effectiveness

None of the interventions in the three studies significantly improved ART adherence or VL outcomes in their primary analyses (all GRADE level 4). SMS text messaging did not significantly improve ART adherence over a 48-week period. Mean adherence (percentage of prescribed doses taken) was 64% in the group that received text messages (p = 0.27 compared to control), 61% in the group that received text messages with the option to respond (p = 0.15 compared to control), and 67% in the control group [51]. The study comparing participant savings accounts to standard care also showed no statistically significant difference in viral suppression between arms. The proportion of participants with a VL < 40 copies/µl at 24 months did not significantly differ by arm − 65.9% in the intervention arm and 63.4% in the control arm [52]. The observational study of intensive adherence counseling also observed low rates of VL suppression post-counseling. Among 192 adolescents included in the study, 117 (60%) had a repeat VL measurement after 3 counseling sessions and 34/117 (29%) achieved a VL < 1000 copies/µl [53].

### Health Services Interventions

#### Study Design, Outcomes, and Participant Eligibility

The four studies evaluating health services interventions included three retrospective cohort studies and one RCT. The study evaluating multi-month ART prescriptions was a retrospective cohort study and used programmatic data to report outcomes of children and adolescents who transitioned to this less intensive model of care after they were deemed clinically stable and ART adherent, defined as having an improving CD4 + cell count or CD4% or a VL < 400 copies/µl and pharmacy pill count > 95%. The primary outcome − VL < 400 copies/µl—was compared between age groups (< 1 year, 1–4 years, 5–9 years, 10–14 years, and 15–19 years) [54]. The group-based intervention study evaluating community-based adherence clubs was a retrospective cohort study among patients who self-reported ART adherence, had been on ART for > 12 months, and had 2 consecutive VL measurements < 400 copies/µl. Outcomes were maintaining a VL < 400 copies/µl and viral rebound at 12 months from enrollment and were compared between age groups (16–24 years and ≥ 25 years) [55]. Since eligibility for both of these studies required established ART adherence and clinical stability, these evaluations aimed to determine if adolescents and youth could maintain a stable status after shifting to a less intensive model of care.

The two studies of health service interventions which evaluated a more intensive model of care—home-based case management in addition to regular clinical care – included one retrospective cohort study and one RCT. The study evaluating the community-based support worker intervention examined a retrospective cohort of adolescents and youth ages 10 to 24 years who were newly diagnosed with HIV and were ART-naïve. Outcomes included mean medication possession ratio (ratio of days of dispensed medication to days between pharmacy visits) and VL < 400 copies/µl assessed at 3 and 5 years from initiating ART. Outcomes were compared to participants who did not receive support over the same 5 year study period [56]. The study evaluating community adolescent treatment supporters was a two-arm RCT among adolescents ages 10 to 15 years who were in care at an HIV care facility and prescribed ART. Participants were randomized to the treatment supporter intervention or standard care and the primary outcome—self-reported ART adherence—was compared by study arm [57].

#### Intervention Effectiveness

Both studies of less intensive models of care—multi-month ART prescriptions [54] and group-based care [55]—showed a relatively high proportion of adolescents and youth maintained a VL < 400 copies/µl over study follow-up (75% and 97.2%, respectively) (GRADE level 2). However in both studies, compared to children and adults, adolescents and youth were at higher risk for viral rebound (i.e. VL > 400 copies/µl). In the study evaluating multi-month prescriptions, at baseline approximately 85% of children (ages 1–9 years) and 80% of adolescents (ages 10–19 years) had a VL < 400 copies/µl. Over 60 months of follow-up with annual VL measurements, this proportion remained steady among children, but decreased among adolescents to approximately 75% [54]. In the study of community-based adherence clubs, the proportion of adolescents (ages 16–24 years) retained in care at 12 months was significantly lower than that observed among adults (ages ≥ 25 years) − 90.9% vs. 94.1%, respectively (p = 0.022). Among those retained in care, the proportion of participants with VL < 400 copies/µl was similar − 97.2% of adolescents and 98.0% of adults (p = 0.194)—but in adjusted analyses, adolescents were at significantly higher risk of experiencing viral rebound compared to adults (adjusted hazard ratio: 2.24; 95% CI 1.0–5.04) [55].

The two studies examining intensified models of care—home-based case management [56, 57]—were the sole studies included in our review which improved ART adherence and viral suppression. The community-based support worker intervention which involved home visits by a lay health support worker, found that 5 years from initiating ART, 81.2% of participants receiving the support intervention achieved a VL < 400 copies/µl compared to 62.8% among those not receiving support (adjusted odds ratio: 0.24; 95% CI: 0.06–1.03; p = 0.055) (GRADE level 4). In the RCT examining the community adolescent treatment supporter intervention which involved home visits from a peer counselor also living with HIV, 12-months from study enrollment, 71.8% of participants in the intervention arm self-reported ART adherence compared to 39.3% receiving standard care (p < 0.05) (GRADE level 3).

### Additional Studies in High Income Countries

There were four additional studies which met inclusion criteria but were conducted in high income settings. Two studies evaluated intensive individual or group counseling in the United States. The Positive Strategies to Enhance Problem-Solving Skills (STEPS) intervention consisted of five one-hour counseling sessions rooted in cognitive-behavioral theory and motivational interviewing skills administered by a master’s or doctoral-level clinician [58]. In a pilot RCT including participants self-reporting < 90% adherence at baseline, 14 participants were randomized to the STEPS intervention or standard care. After 4 months, mean ART adherence among participants randomized to STEPS increased by 13%, and decreased by 26% among participants randomized to standard care, measured by MEMS data. No statistical comparison was conducted for this pilot RCT. The ACCEPT intervention was a group-based educational intervention including topics on stigma, disclosure, healthy relationships, and life planning [59]. In an RCT including 103 adolescents and youth newly diagnosed with HIV, participants in the ACCEPT intervention arm had a 2.33 greater likelihood of self-reported ART adherence at 12 months than those in the control arm (p = 0.005).

Two studies evaluating technology-driven interventions showed statistically significant improvements in VL suppression. In a prospective cohort study conducted in Argentina, an intervention of twice-monthly private messaging through social media was evaluated among adolescents and youth with 2 consecutive VL measurements > 1000 copies/µl. Twenty-two participants were enrolled and at 32 weeks, 64% achieved a VL < 1000 copies/µl [60]. An RCT conducted in the United States included 66 participants ages 14–26 years with a detectable VL at baseline and evaluated an iPhone game designed around themes of ‘fighting’ or ‘destroying’ HIV in the body by taking ART. After 16-weeks, among participants who had newly initiated ART, the decrease in mean log VL of participants in the intervention arm (3.63–0.93) was significantly greater than the decrease in mean log VL of participants in the control arm (3.94–1.53) (p = 0.04) [61].

## Discussion

This systematic review identified seven studies published between 2015 and 2019 which evaluated a patient-level or health service intervention to improve ART adherence among adolescents and youth living with HIV ages 10–24 years in a LMIC. This expands upon a prior review of the same topic examining studies published between 2003 and 2015 [42], none of which were conducted in a LMIC. Among the seven studies in our review, three employed experimental designs appropriately powered to detect intervention effects, and five included a VL outcome, an objective biomarker of adherence [31]. The increase in the number and location of studies observed compared to the earlier review indicates a positive shift of focus to identify interventions to improve outcomes in regions with the greatest HIV burden. Additional high-quality evidence should be emerging soon from four ongoing RCTs we identified, two from sub-Saharan Africa [62–65].

The outcomes of the interventions evaluated indicate that ART adherence and viral suppression among adolescents and youth remain a major challenge. None of the three studies evaluating patient-level interventions, including two RCTs contributing high-quality evidence, showed improvement in either outcome. Adolescents and youth face a variety of barriers to ART adherence, which evolve as they develop physically, emotionally, and socially. Interventions designed to target a single, specific challenge, such as forgetfulness, may be insufficient. For example, the text message intervention was very narrow in scope—no additional counseling was done via text message and the messages were only sent weekly, so did not serve as a daily reminder for taking ART medication. Further research on combination interventions such as incorporating text messaging within a larger package of services is warranted, particularly as such combination intervention strategies have been effective among adults [66, 67].

The majority of studies reviewed focused on how HIV services were delivered to adolescents or youth [54–57]. The two studies on less intensive models of care indicated that adolescents and youth may be less likely to remain clinically stable as they were more likely to experience viral rebound than children or adults in both studies. Additional studies show that among adolescents and youth who do achieve VL suppression, only 50% maintain this for one year [68]. Given adolescence itself is a dynamic period marked by constant development and changes to risk factors, and adolescents and youth are known to have suboptimal viral suppression outcomes, the concept of ‘stable’ adolescents and youth may be misleading.

Conversely, the more intensive HIV care models were the most effective interventions. Home-based case management interventions, which provided additional counseling to identify and address specific barriers which may not be recognized in the clinical setting, showed improved adherence and viral suppression outcomes. Individualized care and treatment within a larger health system may be the most efficient way to identify multiple or evolving challenges and then respond with a custom combination strategy. For example, if an adolescent or youth faces family discrimination, inability to miss school for clinic appointments, and lack of a trusted clinician relationship, the case-management intervention can target this specific barrier combination – family and social isolation, structural challenges of clinic-based care, and poor provider relationship [69, 70].

Several methodological limitations in the studies included should be noted. While community adolescent treatment supporters significantly improved outcomes [57], ART adherence was ascertained through self-report which may be subject to social desirability bias artificially leading to a positive effect. More objective adherence measures such as MEMS, drug levels or VL outcomes could avoid bias. The community-based support worker intervention also showed improved outcomes [56], but assignment to the intervention was not randomized. Patients received the intervention based on availability of support workers in the area, thus unmeasured confounding cannot be ruled out. For example, support workers may have only been available in more developed or easier-to-access locations. Further, as this intervention was evaluated using existing clinical data in a retrospective cohort design, data on the quality and consistency of intervention implementation is lacking, including frequency of home visits, type of support provided, and content of counseling. Lastly, a majority of studies report results just 1-year from implementing the intervention [50–52, 54, 56]. Evaluation of an intervention’s effect over a longer period, particularly for new models of HIV care, could provide information on the long-term durability of an intervention on participant outcomes.

An additional limitation is the inability to identify adolescents and youth who have adequate ART adherence but are not virally suppressed, likely because of the presence of ART drug resistance. For participants who are ART-naïve or who have changed ART regimens, VL outcomes can accurately reflect improved ART adherence, but those who may have acquired ART drug resistance are unlikely to be VL suppressed even with improved ART adherence [71]. Four of the seven studies in this review only report VL outcomes and not an ART adherence measurement and only one is limited to ART-naïve participants [56]. Reporting the ART regimen prescribed could begin to address this limitation since research shows certain regimens are more likely to produce resistance than others [72, 73]. Only one study in this review included data on participants’ prescribed ART regimen [53].

Some interventions implemented in a high income setting improved outcomes among adolescents and youth [58–61] and may be feasibly adapted for use in resource-limited settings. Those utilizing social media or smart phone applications show promise given rapidly increasing access to internet and mobile phones among young people in LMICs [74]. Conversely, implementing interventions involving cognitive behavioral theory and motivational interviewing in high-burden resource-limited settings is unlikely due to the high level of counselor training (i.e. implemented by PhD-level practitioner) and amount of time required for individualized counseling.

Medication-related barriers—side-effects and ease of daily dosing—are critical aspects of adherence and require simplification. We did not include studies which address such barriers through use of alternative regimens or formulations. Use of more potent and/or better tolerated ART regimens such as integrase inhibitors, long-acting and injectable regimens [75–77] have shown promise among adults and should be examined among adolescents and youth. Other medication-related interventions among adolescents and youth such as weekends off regimens have been shown to be non-inferior [78], and single-tablet regimens have achieved significantly higher VL suppression rates than multi-tablet regimens [79]. Medication-related interventions implemented in parallel with health services interventions could significantly and sustainably impact adolescent and youth ART adherence.

## Conclusion

ART adherence and viral suppression outcomes remain a major challenge among adolescents and youth living with HIV in LMICs. Recent studies of interventions to improve ART adherence among this population show inconsistent effects, highlighting the need for additional, innovative approaches which specifically target the needs of adolescents and youth. To date, individually-targeted interventions have not shown significant effects on adherence, but health services interventions which enhance clinic-based care with home-based care, appear promising. There is a clear need for appropriately powered studies examining combination interventions. Standardizing the key outcomes applied across these studies can streamline limited resources, maximize the impact of research, and yield effective interventions to improve health outcomes for adolescents and youth living with HIV.
